# Supplementary material for: Brain songs framework used for discovering the relevant timescale of the human brain
Source: Nat Commun. 2019 Feb 4;10:583. doi: 10.1038/s41467-018-08186-7 (PMC6361902; doi:10.1038/s41467-018-08186-7)
Supplement: Supplementary file 1 — Supplementary Information [file 41467_2018_8186_MOESM1_ESM.pdf]

Supplementary Information

“Brain songs framework used for discovering the relevant timescale of the human brain”

Deco et al.

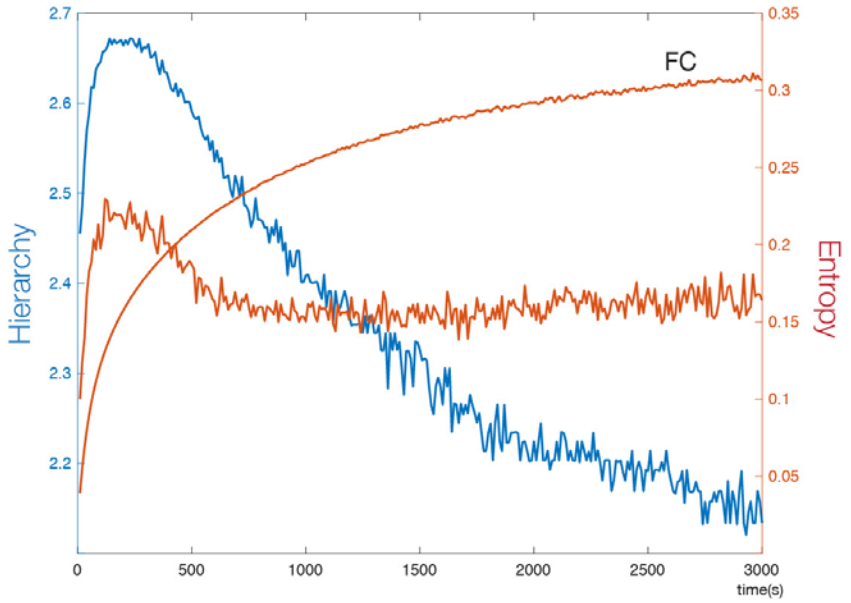

Supplementary Figure 1. Confirming the reliability of the timescale findings in a large, state-of-the-art dataset. *We analysed the reliability of the results in the freely available high-reliability, high-quality Human Connectome Project (HCP) data of 100 unrelated subjects with 15 minutes resting state and a much faster TR of 0.72 seconds (see Methods). The figure shows very similar results to those shown in Figure 3A, where entropy (red line) and hierarchy (blue line) are computed from the whole-brain model fitted to the empirical neuroimaging data in different bin sizes (from 10ms to 3000ms) show a peak in the region of around 200ms.*

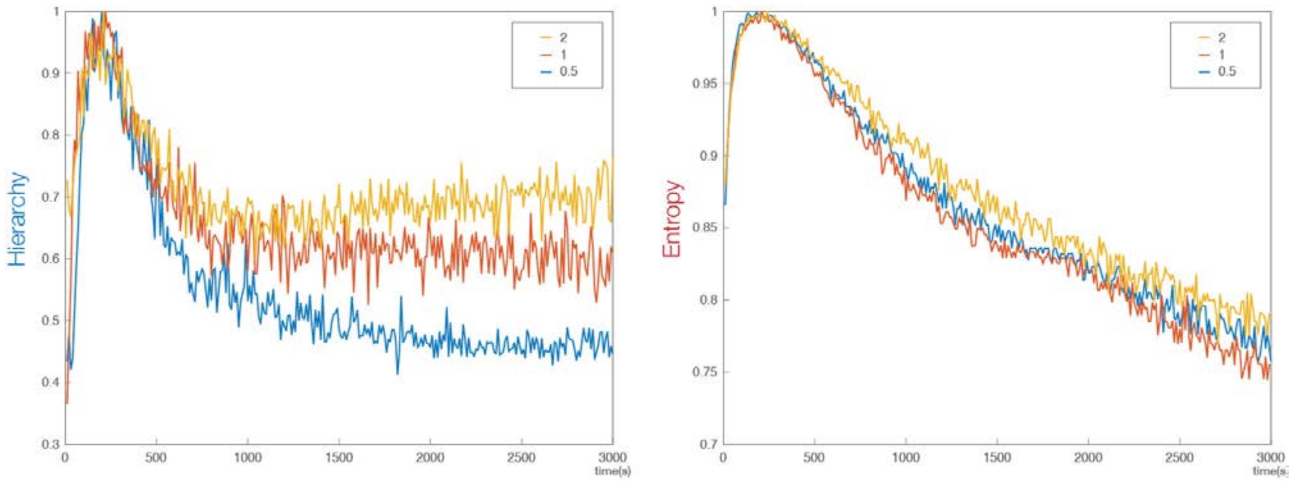

Supplementary Figure 2. Confirming the reliability of the binarisation process. *We ran the full analysis using three different threshold for the binarisation process in order to show that results are threshold independent, as expected given the use of the Poincaré section method. The figure clearly shows the same consistent maximum at around 200ms for entropy and hierarchy for the three thresholds (0.5 – blue line, 1 - red line, and 2 - yellow line - multiplying the standard deviation of the signal). The maxima of the entropy and hierarchy curves are normalised to 1 in order to facilitate the visualisation of the position of the peak. This clearly demonstrates the robustness of the results and the independence of the threshold binarisation.*

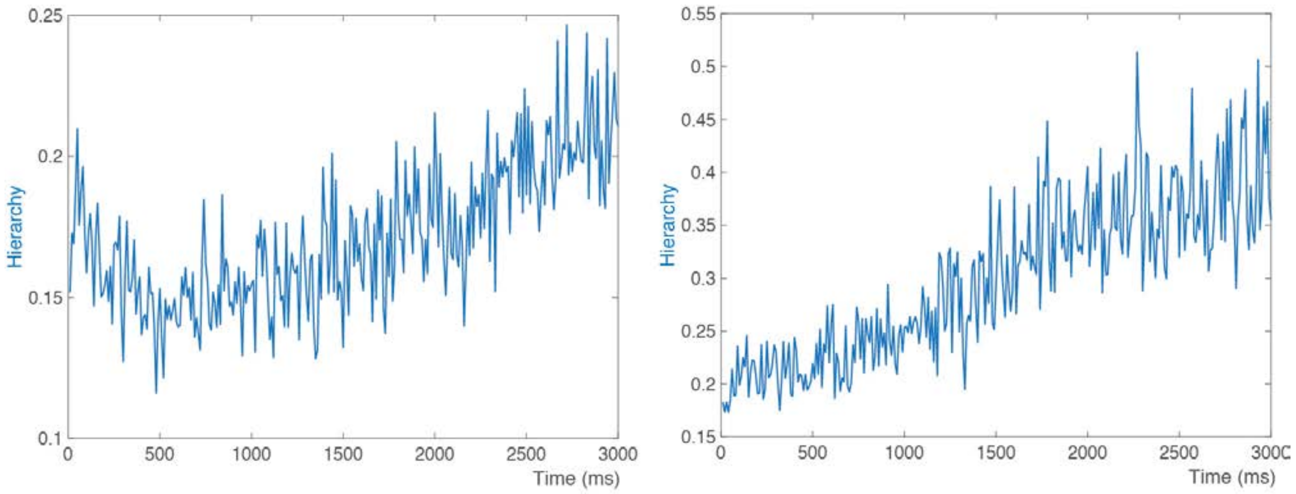

Supplementary Figure 3. Insights into the underlying mechanisms of timescale through changing the local dynamics of whole-brain model. *We investigated the causal dependence of the results on the local regional dynamics of the whole-brain model. The left panel shows the result of changing the ratio of excitation to inhibition at the local regional level and the right panel shows the results of changing the biophysical latencies of the NMDA of the local dynamics to a non-biological value. As can be seen these manipulations break the timescale and remove the peak in the timescale.*

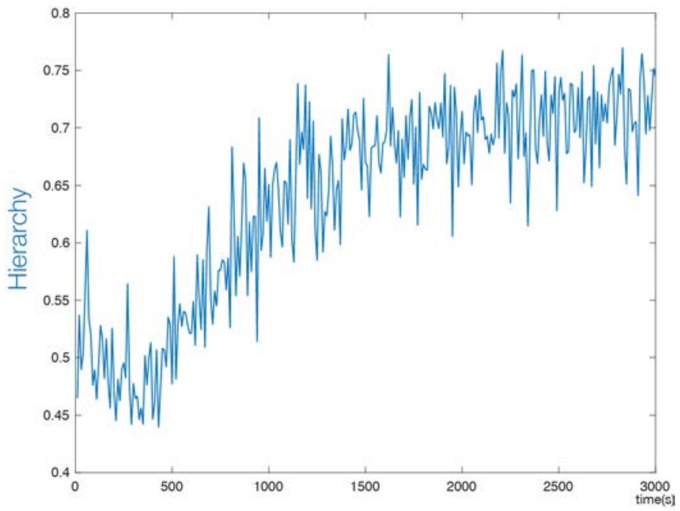

Supplementary Figure 4. Insights into the underlying mechanisms of timescale through manipulating whole-brain model coupling. *We further investigated the causal dependence of the results on the parameters of the whole-brain model by manipulating the whole-brain coupling. We focused on measuring hierarchy, which is the most sensitive measure for measuring timescale. We show the effects of shifting the working point of the whole-brain model away from optimal (from G=2.18 to G=0.25). The figure shows that the hierarchy no longer shows a peak in timescale at 200ms. This clearly shows that only the realistic whole-brain model fitting the empirical data is showing the relevant timescale of 200ms (shown in Figures 3 and 6).*

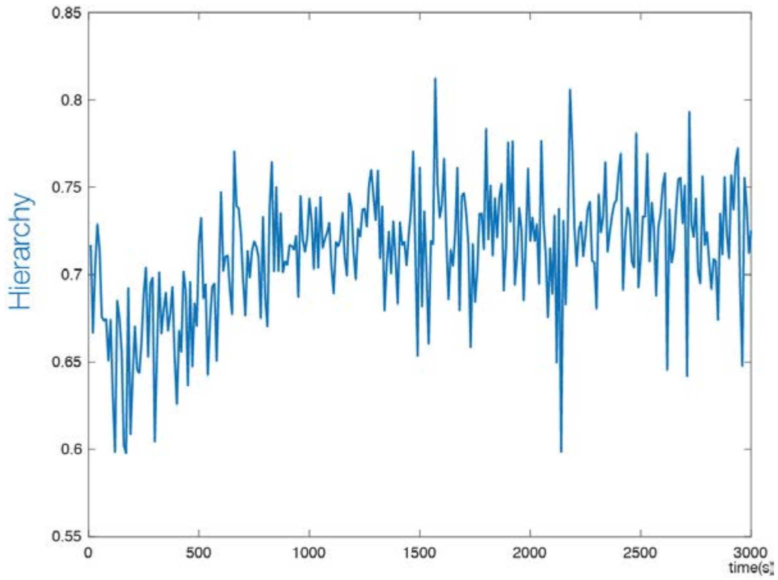

Supplementary Figure 5. Further insights into the mechanisms underlying timescale through manipulating the underlying connectome of the model. *Additional investigations of the timescale as measured with the sensitive hierarchy measure were carried out by damaging the highest coupled edges in the whole-brain network (top 20%). The figure shows that the timescale is significantly affected by this manipulation, similar to the results shown in Supplementary Figure 3 following non-optimal whole-brain coupling. In summary, these results suggest that the relevant timescale emerges from the exact empirical structural connectivity coupling of the human brain.*
