# Supplementary material for: Brain songs framework used for discovering the relevant timescale of the human brain
Source: Nat Commun. 2019 Feb 4;10:583. doi: 10.1038/s41467-018-08186-7 (PMC6361902; doi:10.1038/s41467-018-08186-7)
Supplement: Supplementary file 3 — Reporting summary [file 41467_2018_8186_MOESM3_ESM.pdf]

nature research

Corresponding author(s): Deco, Kringelbach

Reporting Summary

Nature Research wishes to improve the reproducibility of the work that we publish. This form provides structure for consistency and transparency in reporting. For further information on Nature Research policies, see Authors & Referees and the Editorial Policy Checklist.

Statistical parameters

When statistical analyses are reported, confirm that the following items are present in the relevant location (e.g. figure legend, table legend, main text, or Methods section).

n/a	Confirmed	
☐	☒	The exact sample size (n) for each experimental group/condition, given as a discrete number and unit of measurement
☐	☒	An indication of whether measurements were taken from distinct samples or whether the same sample was measured repeatedly
☒	☐	The statistical test(s) used AND whether they are one- or two-sided<br>*Only common tests should be described solely by name; describe more complex techniques in the Methods section.*
☐	☒	A description of all covariates tested
☒	☐	A description of any assumptions or corrections, such as tests of normality and adjustment for multiple comparisons
☒	☐	A full description of the statistics including central tendency (e.g. means) or other basic estimates (e.g. regression coefficient) AND variation (e.g. standard deviation) or associated estimates of uncertainty (e.g. confidence intervals)
☒	☐	For null hypothesis testing, the test statistic (e.g. F, t, r) with confidence intervals, effect sizes, degrees of freedom and P value noted<br>*Give P values as exact values whenever suitable.*
☒	☐	For Bayesian analysis, information on the choice of priors and Markov chain Monte Carlo settings
☒	☐	For hierarchical and complex designs, identification of the appropriate level for tests and full reporting of outcomes
☒	☐	Estimates of effect sizes (e.g. Cohen's d, Pearson's r), indicating how they were calculated
☒	☐	Clearly defined error bars<br>*State explicitly what error bars represent (e.g. SD, SE, CI)*

Our web collection on statistics for biologists may be useful.

Software and code

Policy information about availability of computer code

Data collection	Standard MRI and MEG scanner software
Data analysis	Standard imaging tools for preprocessing FSL (FMRIB Software Library), FreeSurfer, and the Connectome Workbench software. Custom-made MATLAB scripts were used for whole-brain modelling. We will make these available upon request.

For manuscripts utilizing custom algorithms or software that are central to the research but not yet described in published literature, software must be made available to editors/reviewers upon request. We strongly encourage code deposition in a community repository (e.g. GitHub). See the Nature Research guidelines for submitting code & software for further information.

Data

Policy information about availability of data

All manuscripts must include a data availability statement. This statement should provide the following information, where applicable:

- Accession codes, unique identifiers, or web links for publicly available datasets
- A list of figures that have associated raw data
- A description of any restrictions on data availability

The code and multimodal neuroimaging data from the experiment are available upon request.

Field-specific reporting

Please select the best fit for your research. If you are not sure, read the appropriate sections before making your selection.

☒ Life sciences ☐ Behavioural & social sciences ☐ Ecological, evolutionary & environmental sciences

For a reference copy of the document with all sections, see nature.com/authors/policies/ReportingSummary-flat.pdf

Life sciences study design

All studies must disclose on these points even when the disclosure is negative.

Sample size	We used data from two populations. One group of 16 participants from Aarhus, Denmark and one group of 100 unrelated participants from the public available database from the Human Connectome Project (HCP) from the WU-Minn HCP Consortium.
Data exclusions	N/A
Replication	Findings from Dataset 1 were replicated in Dataset 2
Randomization	N/A
Blinding	N/A

Reporting for specific materials, systems and methods

Materials & experimental systems

n/a	Involved in the study
☒	☐ Unique biological materials
☒	☐ Antibodies
☒	☐ Eukaryotic cell lines
☒	☐ Palaeontology
☒	☐ Animals and other organisms
☐	☒ Human research participants

Methods

n/a	Involved in the study
☒	☐ ChIP-seq
☒	☐ Flow cytometry
☐	☒ MRI-based neuroimaging

Human research participants

Policy information about studies involving human research participants

Population characteristics	We used data from two populations. One group of 16 participants from Aarhus, Denmark and one group of 100 unrelated participants from the public available database from the Human Connectome Project (HCP) from the WU-Minn HCP Consortium.
Recruitment	Dataset1: The online recruitment system at Aarhus University helped to recruit all 16 healthy right-handed participants (11 men and 5 women, mean age: 24.75+/-2.54). We screened participants and excluded those with psychiatric or neurological disorders (or a history thereof) from participation in this study.<br>Dataset 2: The data set used for this investigation was selected from the March 2017 public data release from the Human Connectome Project (HCP) where we chose the sample of 100 unrelated participants (54 females, 46 males, mean age=29.1+/-3.7 years). This subset of participants provided by HCP ensures that they are not family relatives. This criterion was important to exclude possible identifiability confounds and the need for family-structure co-variables in the analyses.

Magnetic resonance imaging

Experimental design

Design type	Resting state and task design
Design specifications	Dataset 1: We collected approximately seven minutes of resting state data for 16 subjects.<br>Dataset 2: 100 unrelated HCP participants. The HCP website (http://www.humanconnectome.org/) provides the full details of participants, the acquisition and preprocessing of the data.

Behavioral performance measures: N/A

Acquisition

Imaging type(s): functional and diffusion MRI

Field strength: 3T

Sequence & imaging parameters: Dataset 1: The parameters for the structural MRI T1 scan used a voxel size of 1 mm3; reconstructed matrix size 256x256; echo time (TE) of 3.8 ms and repetition time (TR) of 2300 ms. The resting-state fMRI data were collected using whole-brain echo planar images (EPI) with TR = 3030 ms, TE = 27 ms, flip angle = 90˚, reconstructed matrix size = 96x96, voxel size 2x2 mm with slice thickness of 2.6 mm and a bandwidth of 1795 Hz/Px. We collected approximately seven minutes of resting state data per subject.
Dataset 2: The 100 HCP participants were scanned on a 3-T connectome-Skyra scanner (Siemens). We used one resting state fMRI acquisition of approximately 15 minutes acquired on the same day, with eyes open with relaxed fixation on a projected bright cross-hair on a dark background. The HCP website (http://www.humanconnectome.org/) provides the full details of participants, the acquisition and preprocessing of the data.

Area of acquisition: Whole brain

Diffusion MRI: ☒ Used ☐ Not used

Parameters: dMRI data was acquired using TR = 9000 ms, TE = 84 ms, flip angle = 90˚, reconstructed matrix size of 106x106, voxel size of 1.98x1.98 mm with slice thickness of 2 mm and a bandwidth of 1745 Hz/Px. Furthermore, the data were collected with 62 optimal nonlinear diffusion gradient directions at b=1500 s/mm2. Approximately one non-diffusion weighted image (b=0) per 10 diffusion weighted images was acquired.

Preprocessing

Preprocessing software: Standardized methods using FSL (FMRIB Software Library), FreeSurfer, and the Connectome Workbench software

Normalization: FLIRT

Normalization template: MNI152

Noise and artifact removal: The head motion parameters were regressed out and structured artefacts were removed by ICA+FIX processing (Independent Component Analysis followed by FMRIB's ICA-based X-noiseifier).

Volume censoring: N/A

Statistical modeling & inference

Model type and settings: Whole-brain model

Effect(s) tested: Spatiotemporal structure

Specify type of analysis: ☒ Whole brain ☐ ROI-based ☐ Both

Statistic type for inference (See Eklund et al. 2016): N/A

Correction: N/A

Models & analysis

n/a	Involved in the study
☐	☒ Functional and/or effective connectivity
☒	☐ Graph analysis
☒	☐ Multivariate modeling or predictive analysis

Functional and/or effective connectivity: Pearson correlation
